# Probing Metal Ion Discrimination in a Protein Designed to Bind Uranyl Cation With Femtomolar Affinity

**DOI:** 10.3389/fmolb.2019.00073

**Published:** 2019-08-27

**Authors:** Marie Hoarau, Karl J. Koebke, Zhan Chen, E. Neil G. Marsh

**Affiliations:** ^1^Department of Chemistry, University of Michigan, Ann Arbor, MI, United States; ^2^Department of Biological Chemistry, University of Michigan, Ann Arbor, MI, United States

**Keywords:** protein engineering, metalloproteins, metal ion selectivity, helical bundle proteins, EPR spectroscopy

## Abstract

The design of metal-binding sites in proteins that combine high affinity with high selectivity for the desired metal ion remains a challenging goal. Recently, a protein designed to display femtomolar affinity for ${\text{UO}}_{2}^{2+}$, dubbed “Super Uranyl-binding Protein” (SUP), was described, with potential applications for removing ${\text{UO}}_{2}^{2+}$ in water. Although it discriminated most metal ions present in seawater, the protein showed a surprisingly high affinity for Cu^2+^ ions. Here, we have investigated Cu^2+^ binding to SUP using a combination of electron paramagnetic resonance, fluorescence and circular dichroism spectroscopies. Our results provide evidence for two Cu^2+^ binding sites on SUP that are distinct from the ${\text{UO}}_{2}^{2+}$ binding site, but one of which interferes with ${\text{UO}}_{2}^{2+}$ binding. They further suggest that in solution the protein's secondary structure changes significantly in response to binding ${\text{UO}}_{2}^{2+}$; in contrast, the crystal structures of the apo- and holo-protein are almost superimposable. These results provide insights for further improving the selectivity of SUP for ${\text{UO}}_{2}^{2+}$, paving the way toward protein-based biomaterials for decontamination and/or recovery of uranium.

## Introduction

Proteins have evolved to bind metal ions with remarkable selectivity. This is achieved by controlling both the chemical nature and the geometry of the coordinating ligands at the metal binding site (Montes-Bayón and Blanco-González, 2016). As a result, proteins also exercise exquisite control over the reactivity of the metals they bind, for example fine-tuning properties such as Lewis acidity, oxidation state, and redox potential. However, biology utilizes a relatively small subset of metals and hence there has been considerable interest in designing proteins that bind non-biological metal ions, such as those in the lanthanide and actinide series (Le Clainche et al., 2003; Barak et al., 2006; Le Clainche and Vita, 2006; Wegner et al., 2009; Chakraborty et al., 2011; Plegaria et al., 2015; Starck et al., 2015; Brulfert et al., 2016). The design of proteins (and nucleic acids) that bind uranium has been of particular interest, given that this element is an essential component in nuclear weapons and nuclear reactors (Handley-Sidhu et al., 2010). Potential applications of uranium-binding proteins include biosensing and bio-remediation of uranium-contaminated environments that may result from the use of depleted uranium in munitions and from uranium processing associated with nuclear weapons and nuclear fuel manufacture (Bhalara et al., 2014; Newsome et al., 2014; Xie et al., 2019).

Recently, a small (80 residue) α-helical protein was described that had been engineered to bind uranyl cation, ${\text{UO}}_{2}^{2+}$, the predominant form of uranium in the environment, with remarkably high, femtomolar affinity. This protein, dubbed Super Uranyl-binding Protein (SUP), also exhibits very high selectivity constants against other environmentally more abundant metal ions. These ranged from 10^3^ to 10^7^ for a series of the most common metal ions found in sea water (Zhang et al., 2014), suggesting that the protein might have potential for extracting ${\text{UO}}_{2}^{2+}$ from seawater. SUP has also subsequently been incorporated into protein hydrogels (Kou et al., 2017a,b) and 2D protein multilayers (Zhang et al., 2018) with high ${\text{UO}}_{2}^{2+}$ adsorption capacity. These materials could be used for the decontamination of depleted uranium, often found in groundwaters in former conflict areas or for the development of highly sensitive ${\text{UO}}_{2}^{2+}$ biosensors.

**Figure 1:**
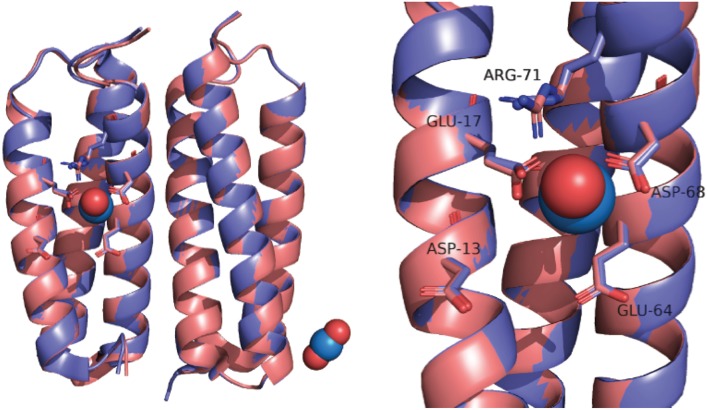
Overlay of SUP crystal structures obtained with (pink) and without ${\text{UO}}_{2}^{2+}$ bound (purple).

In part, the selectivity of SUP derives from the atypical geometry of the uranyl cation which is a linear molecule. SUP was designed to bind ${\text{UO}}_{2}^{2+}$ with pentagonal bipyramid geometry: five equatorial oxygen ligands to uranium are supplied by bidentate coordination of Glu17 and Asp68 and a water molecule, whereas the two axial positions are occupied by the oxo ligands of uranyl (Figure 1). An important stabilizing interaction is provided by Arg71, which supplies a hydrogen-bond to one of the axial oxo-ligands. However, MD simulations suggest a slightly different coordination sphere from that seen in the crystal structure, with Glu64 and Asp13 binding ${\text{UO}}_{2}^{2+}$ in a monodentate fashion, while Glu17 and Asp68 bind ${\text{UO}}_{2}^{2+}$ in an alternate monodentate/bidentate fashion (Odoh et al., 2014).

An oxygen-rich coordination sphere is very typical of ${\text{UO}}_{2}^{2+}$-protein complexes, due to the “hard” nature of the ${\text{UO}}_{2}^{2+}$ cation and is in contrast to the binding of transition metal ions, which invariably include nitrogen or sulfur ligands (Van Horn and Huang, 2006; Carugo, 2018).

With this in mind, we were intrigued by the reported strong competition for SUP binding exhibited by Cu^2+^. It was found that the presence of a ~10^3^-fold excess of Cu^2+^ completely prevented ${\text{UO}}_{2}^{2+}$ from binding SUP (Zhang et al., 2014). Indeed Cu^2+^ was more effective at competing for binding than VO^2+^, Pb^2+^, or Mn^2+^, which required between 10^4^- and 10^5^-fold excesses of metal ions to effectively displace ${\text{UO}}_{2}^{2+}$ from the binding site. Cu^2+^ concentrations in seawater range between 0.004 and 1.6 μM (Campbell et al., 2014), indicating that Cu-binding could significantly impede the ability of SUP to bind ${\text{UO}}_{2}^{2+}$ dissolved in the ocean.

In this study, we have re-evaluated the binding of Cu^2+^ to SUP and its ability to compete with ${\text{UO}}_{2}^{2+}$. Our results indicate that Cu^2+^ binds at two sites on the protein, but each is distinct from the ${\text{UO}}_{2}^{2+}$-binding site so that displacement of ${\text{UO}}_{2}^{2+}$ by Cu^2+^ may occur by an indirect mechanism rather than simple competition. This view is supported by the observation of changes in the CD spectrum of SUP that occurs upon ${\text{UO}}_{2}^{2+}$ binding, which suggest that conformational changes to SUP are involved in binding ${\text{UO}}_{2}^{2+}$.

## Results and Discussion

### Structural Changes to SUP Accompanying ${\text{UO}}_{2}^{2+}$ Binding

The crystal structure of SUP has been solved both with ${\text{UO}}_{2}^{2+}$ bound and in the absence of metal ions (PDB 4FZO and 4FZP, respectively). The structures are almost superimposable, arguing that very little structural rearrangement occurs upon binding ${\text{UO}}_{2}^{2+}$ (Figure 1). However, these structures could only be obtained at pH 4, and no structural information is available at physiological pH or at pH 8.9, for which ${\text{UO}}_{2}^{2+}$ binding affinity was determined.

**Figure 2:**
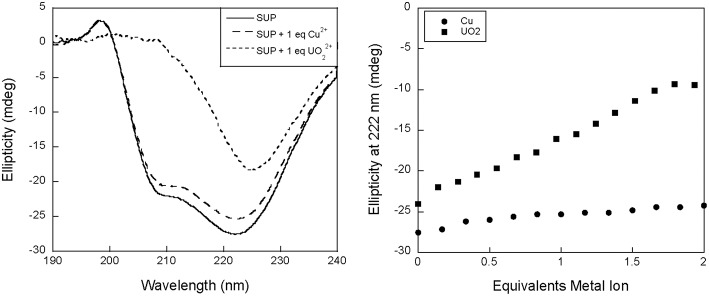
Changes to the circular dichroism spectrum of SUP in the presence of Cu^2+^ and ${\text{UO}}_{2}^{2+}$. **(Left)** CD spectra of SUP in absence and presence of metal ions. **(Right)** corresponding titration curves.

We therefore proposed to study ${\text{UO}}_{2}^{2+}$ binding to SUP at pH 7.5 using circular dichroism (CD). Surprisingly, preliminary measurements revealed a significant change in the CD spectrum of SUP upon addition of metals. Apo-SUP possesses a CD spectrum characteristic of an extensively α-helical protein with minima at 208 and 222 nm; however, as shown in Figure 2, the addition of 1 equivalent of ${\text{UO}}_{2}^{2+}$ to SUP resulted in an almost complete loss of the 208 nm band. This observation suggested that SUP may undergo changes to its structure upon binding metal ions. In particular, changes in the ratio of ellipticities at 222 and 208 nm are indicative of a change in the super-helical pitch of the helical bundle proteins such as SUP (Banerjee and Sheet, 2017).

**Figure 3:**
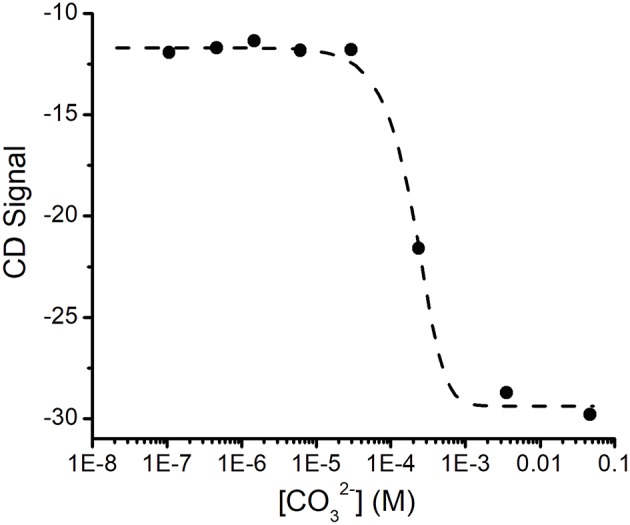
Determination of the K_d_ for ${\text{UO}}_{2}^{2+}$ binding SUP by competition with ${\text{CO}}_{3}^{2-}$. Increasing concentrations of ${\text{CO}}_{3}^{2-}$ ion compete for ${\text{UO}}_{2}^{2+}$ and the displacement of ${\text{UO}}_{2}^{2+}$ was followed by changes in the CD spectrum of SUP. Experimental points were fitted using Dynafit (see Materials and Methods for details).

The changes in the CD spectrum that accompanied ${\text{UO}}_{2}^{2+}$ binding allowed us to measure the dissociation constant for this cation (Figure 3). Previous studies had used the colorimetric metal chelating agent Arsenazo III to determine ${\text{UO}}_{2}^{2+}$ concentrations, which necessitated the separation of SUP-${\text{UO}}_{2}^{2+}$ from free ${\text{UO}}_{2}^{2+}$ by ultrafiltration (Zhang et al., 2014). However, in our hands this assay proved difficult to reproduce and time consuming. In contrast, we were able to establish the dissociation constant for ${\text{UO}}_{2}^{2+}$ quite straightforwardly by measuring the changes in ellipticity at fixed SUP and ${\text{UO}}_{2}^{2+}$ concentrations as a function of increasing concentrations of Na_2_CO_3_ (Figure 3), which is a competitive chelator of ${\text{UO}}_{2}^{2+}$. Fitting the data as described in the Materials and Methods section yielded a K_d_ of ~0.4 fM at pH 7.9. This may be compared with the previously reported K_d_ of 7.4 fM obtained by the Arsenazo III method at pH 8.9 (Zhang et al., 2014). Importantly, Zhang et al. have previously reported a strong pH sensitivity of ${\text{UO}}_{2}^{2+}$ binding affinity, with a reported 0.2 nM affinity at pH 6.0 measured by Arsenazo III method using DGA ligand (Zhang et al., 2014). The difference in the two K_d_ measurements likely rises from differences in the experimental conditions used (notably pH), together with the fact that the calculated affinity of the protein for ${\text{UO}}_{2}^{2+}$ is rather sensitive to small differences in ${\text{CO}}_{3}^{2-}$ concentrations due to the presence of multiple, competing ${\text{UO}}_{2}^{2+}$(${\text{CO}}_{3}^{2-}$)_n_ species. Therefore, given the different methods and conditions by which these K_d_ measurements were obtained, we consider the results to be in reasonable agreement.

### Stoichiometry of Cu(II) Binding to SUP

**Figure 4:**
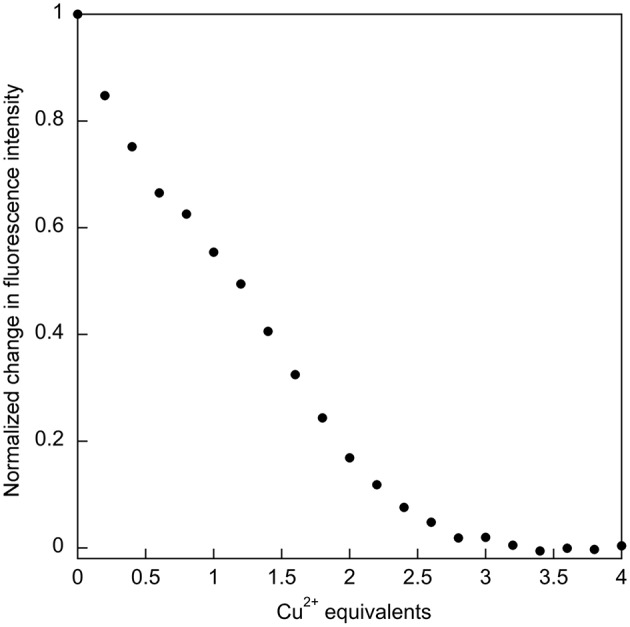
Titration of SUP with CuSO_4_ with binding followed by quenching of tyrosine fluorescence (λ_ex_ = 260 nm; λ_em_ = 305 nm), indicates that two equivalents of Cu^2+^ bind.

The relatively high affinity of SUP for Cu^2+^ had previously been inferred from the ability of this metal ion to compete with ${\text{UO}}_{2}^{2+}$ binding. It was reported that the presence of a 1,000-fold excess of Cu^2+^ was sufficient to prevent ${\text{UO}}_{2}^{2+}$ from binding SUP (Zhang et al., 2014). This competitive effect of Cu^2+^ was much stronger than, for example, VO^2+^ and Ca^2+^, ions that are expected to have coordination preferences similar to ${\text{UO}}_{2}^{2+}$. We therefore decided to examine Cu^2+^ binding to SUP directly. We found that Cu^2+^ binding could be followed by monitoring changes in fluorescence due to the four Tyr residues in the protein. A 5 μM solution of the protein in HEPES buffer, pH 7.5, was titrated with increasing amounts of CuSO_4_ and the fluorescence signal at 305 nm was recorded. The bi-phasic nature of the titration curve (Figure 4) suggested that SUP binds two equivalents of Cu^2+^ at sites that possess different affinities for the metal. This observation clearly implies that at least one of the Cu^2+^ binding sites must be distinct from the ${\text{UO}}_{2}^{2+}$ binding site.

### EPR Spectroscopic Studies on Cu^2+^ Binding to SUP

**Figure 5:**
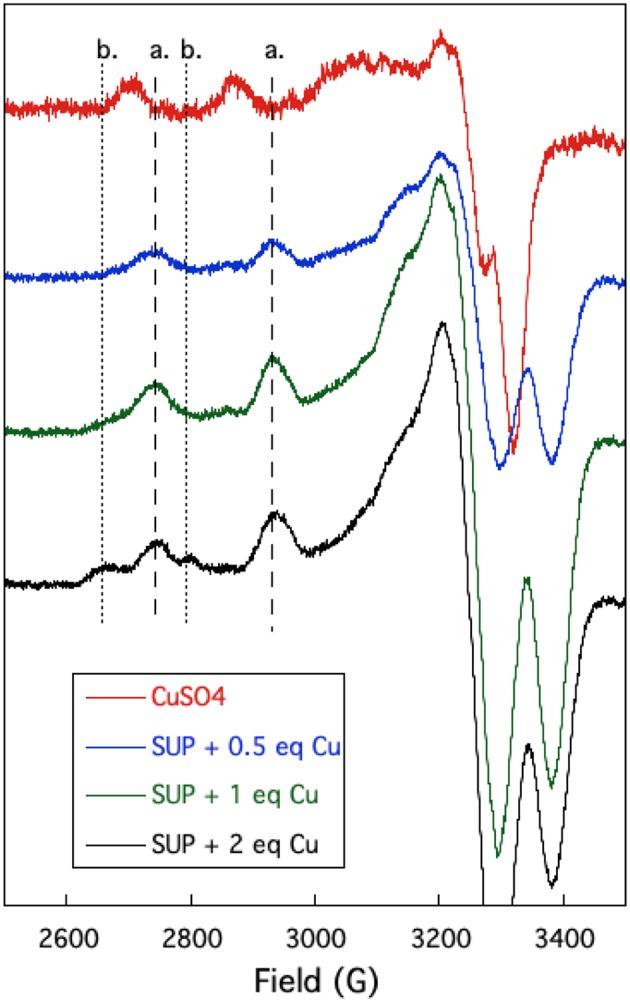
Changes in the EPR spectrum of Cu^2+^ in the presence of SUP. The spectra indicate the presence of two distinct Cu^2+^ binding sites.

The oxygen-rich binding site for ${\text{UO}}_{2}^{2+}$ would seem to be a poor binding site for Cu^2+^ which is a much softer cation. Indeed, Cu-binding sites in proteins invariably utilize at least one or more nitrogen or sulfur atoms as ligands to the metal (Andreini et al., 2008). EPR provides a sensitive method to probe the binding of copper to proteins as the A_//_ and *g*_//_ values are diagnostic for the ligands to the metal (Garribba and Micera, 2006). EPR spectra of Cu^2+^ were recorded in the presence of increasing stoichiometries of SUP in Tris-HCl buffer, pH 7.5 at 150 K. Comparison of spectra provided further evidence for two Cu^2+^ binding sites in SUP. Starting from free Cu^2+^ in solution (Figure 5, red curve), addition of 1 eq SUP gave rise to one set of hyperfine coupling bands, corresponding to the coordination of Cu^2+^ on SUP, denoted as site a (Figure 5, green curve). The addition of a second equivalent of SUP gives rise to a second set of hyperfine coupling bands, corresponding to a second binding site along SUP denoted as site b (Figure 5, black curve), which differs from site a and free Cu^2+^ signals. These data indicate that Cu^2+^ binds to site a first, and then b. The data are consistent with the two-site binding model indicated by the fluorescence studies.

The hyperfine coupling constants and *g* values for site a and site b were calculated from the spectra and are given in Table 1. Comparison of the hyperfine coupling constants obtained for sites a and b with Peisach-Blumberg correlation tables (Peisach and Blumberg, 1974) indicates that the ligands constituting site a are all N-donors, whereas site b is consistent with a 2N_2_O environment.

**Table 1:** EPR parameters for the two binding sites of Cu^2+^ in SUP.

	**A_**//**_ (10^**−4**^ cm^**−1**^)**	**g_**//**_**	**xN_**x**_O**
**Site a**	177	2.207	4N
**Site b**	143	2.327	2N_2_O

This is consistent with statistical survey data that shows that virtually all Cu(II) proteins use at least one N-donor ligand to bind Cu^2+^, while only 21% of them use at least one O-donor ligand (Andreini et al., 2008). This further strengthens the hypothesis that Cu^2+^ is binding SUP through a different set of ligands from ${\text{UO}}_{2}^{2+}$. In their 2014 paper, Odoh et al. performed MD simulations on the ${\text{UO}}_{2}^{2+}$ binding site in order to evaluate a possible binding of Cu^2+^(Odoh et al., 2014). Their results concluded that Cu^2+^ could adopt a 6-O coordination, binding to Asp13 and 68, Glu17 and 64, and with one water molecule. However, a mismatch was detected between selectivity values obtained from this simulation and experimental data, showing that other parts of the protein might be to consider to fully model the SUP-Cu^2+^ interaction.

**Figure 6:**
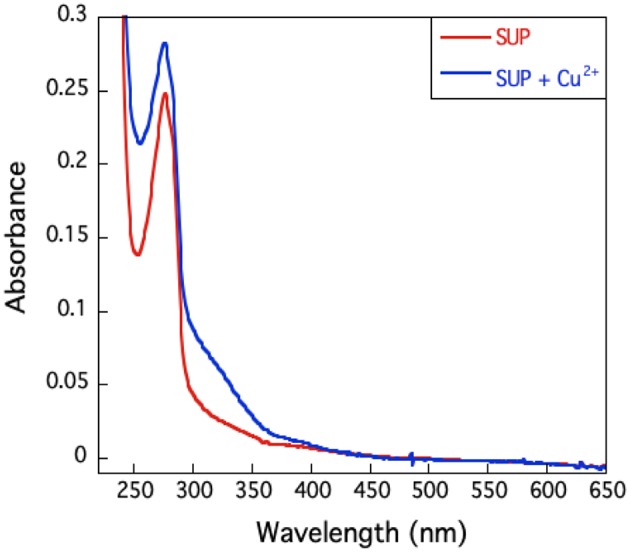
U.V.-visible spectrum of SUP in presence and absence of Cu^2+^. The absence of an absorption band around 600 nm excludes the presence of a type I copper site.

The UV-visible spectrum of SUP complexed with Cu^2+^ (Figure 6) shows no high intensity absorptions at wavelengths longer than 350 nm, which would be typical of a Type I copper protein. Taken together with the EPR hyperfine coupling, this indicates that SUP should be categorized as a Type II Cu protein with no sulfur ligation (Adman, 1991).

**Figure 7:**
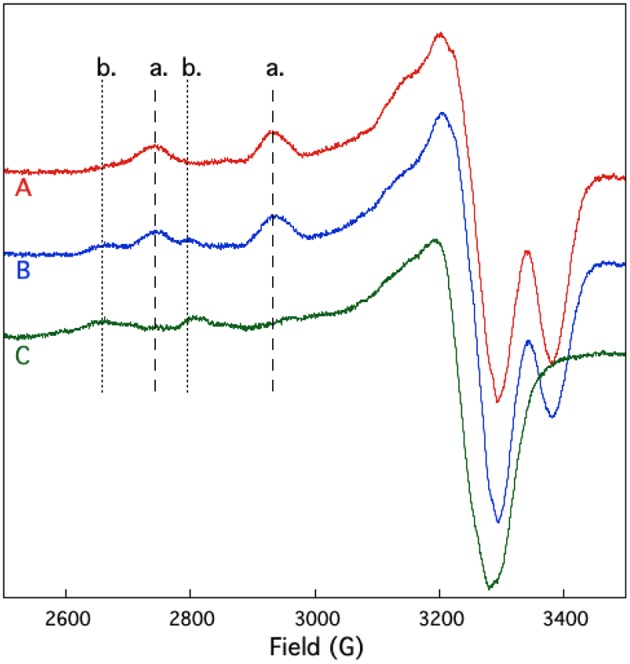
Competition for ${\text{UO}}_{2}^{2+}$ and Cu^2+^ binding to SUP investigated by EPR spectroscopy. Spectrum A: pre-formed SUP-Cu^2+^ complex (1:1 stoichiometry). Spectrum B: pre-formed SUP-${\text{Cu}}_{2}^{2+}$ (1:2 stoichiometry), showing both hyperfine coupling bands for site a and site b. Spectrum C: pre-formed SUP-Cu^2+^complex (1:1 stoichiometry) after addition of 1 equivalent of ${\text{UO}}_{2}^{2+}$, showing that Cu^2+^ is displaced from site a by ${\text{UO}}_{2}^{2+}$ and re-bound at site b.

EPR spectroscopy was also used to study the competition between ${\text{UO}}_{2}^{2+}$ and Cu^2+^ binding to SUP. If ${\text{UO}}_{2}^{2+}$ shares coordinating residues with Cu^2+^, addition of ${\text{UO}}_{2}^{2+}$ to the pre-formed [SUP-Cu_2_] complex should lead to a release of Cu^2+^ in solution, whereas preloading SUP with ${\text{UO}}_{2}^{2+}$ should prevent Cu^2+^ from binding. The results of this experiment are shown in Figure 7. SUP loaded with one equivalent of Cu^2+^ exhibits an EPR spectrum in which copper is bound at the site. However, addition of one equivalent of ${\text{UO}}_{2}^{2+}$ converts the spectrum to that of Cu^2+^ binding to the b site. These data indicate that ${\text{UO}}_{2}^{2+}$ displaces Cu^2+^ from the high affinity a site, causing it to move to the lower affinity b site. The copper a site either shares some metal-coordinating residues in common with the ${\text{UO}}_{2}^{2+}$ site, or the two metal-binding sites are in sufficiently close proximity that ${\text{UO}}_{2}^{2+}$ and Cu^2+^ are unable to bind simultaneously.

## Conclusions

The anomalous affinity of SUP for Cu^2+^ ions, compared with other transition metal ions, may be explained by the fact that Cu^2+^ appears to bind at two sites on the protein. The initial studies of SUP measured the relative affinities of metal ions by examining their ability to displace ${\text{UO}}_{2}^{2+}$ from the protein, rather than examining their binding directly. Based on our EPR data and the coordination environment of other well-characterized Cu-binding proteins (Rubino and Franz, 2012) it seems very unlikely that Cu^2+^ directly competes for the ${\text{UO}}_{2}^{2+}$ binding site. Rather the data suggest that Cu^2+^ indirectly interferes with ${\text{UO}}_{2}^{2+}$ binding. This could occur either by distorting the binding site or possibly competing for one of the protein side-chains that ligate ${\text{UO}}_{2}^{2+}$. These studies highlight an important problem designing proteins that bind metal ions with both high affinity and selectivity, i.e., that metal ions may bind adventitiously to the protein at unintended sites or recruit ligands from the designed binding site that interfere with binding of the intended metal ion.

Interestingly, we observed a significant change in the CD spectrum of SUP that accompanies ${\text{UO}}_{2}^{2+}$ binding. This allowed us to measure the K_d_ for ${\text{UO}}_{2}^{2+}$ binding far more easily than the previously published assay that involved separating protein-bound and free ${\text{UO}}_{2}^{2+}$ and then determining free ${\text{UO}}_{2}^{2+}$ using a chemical test. Also significant, the changes to the CD spectrum suggest that the ${\text{UO}}_{2}^{2+}$-SUP complex may have a different secondary structure from the apo-protein at pH 7.5, possibly associated with a change in the pitch of the alpha-helical bundle. This is of interest because a comparison of the crystal structures of SUP with and without ${\text{UO}}_{2}^{2+}$ bound at pH 4 revealed very little alteration of the protein's structure (Zhang et al., 2014; Figure 1). Further studies into the solution structure of SUP are in progress to better define how ${\text{UO}}_{2}^{2+}$ binding alters the structure of the protein. These studies may help to explain the remarkably high affinity of SUP for this unusual metal-oxo cation.

## Materials and Methods

### Protein Expression and Purification

A *E. coli* codon-optimized gene encoding SUP with an N-terminal 6-His tag followed by a TEV protease cleavage site was commercially synthesized and introduced into the expression vector pET28a by standard methods. This construct was used to transform *E. coli* BL21 DE3 cells by standard methods. Cells were cultured in 1 L of LB medium containing kanamycin 50 mg/L and grown at 37°C until reaching OD_600_ 0.8–1. Protein expression was induced by addition of IPTG, 0.3 mM final concentration, and the cells allowed to grow overnight at 22°C.

Cells were harvested by centrifugation and the cell pellet was resuspended in lysis buffer (Tris-HCl pH 7.5 10 mM, NaCl 300 mM, TCEP 1 mM, protease inhibitor) using 4 mL of buffer per gram of cell paste. Urea was added to a final concentration of 2 M and the cells lysed by sonication, using 10 s pulses followed by a 20 s pause for a total time of 30 min. The lysate was then clarified by centrifugation (16,000 rpm, 45 min, 4°C) and slowly loaded onto a HisTrap column pre-equilibrated with buffer A (Tris-Cl pH 7.5 10 mM, NaCl 300 mM) at 4°C. The column was washed extensively with buffer A, followed by washing with 20% buffer B (Tris pH 7.5 10 mM, NaCl 300 mM, imidazole 500 mM) to remove non-specifically bound proteins. SUP was then eluted from the column by washing with 70% buffer B.

Fractions containing SUP were dialyzed against buffer A overnight and the 6-His-tag was then cleaved by addition of 10 μL TEV protease, followed by incubation at RT for 12 h. The His-tag and any uncleaved protein were removed by incubation with Ni-NTA beads overnight. TEV and remaining contaminant proteins were precipitated by heating the samples at 70°C for 30 min followed by centrifugation (12,000 rpm, 10 min, RT) to remove precipitated contaminants. Finally, the sample was concentrated and then desalted using a Superdex 200 10/30 column, equilibrated in SEC buffer (HEPES pH 7.5 20 mM, NaCl 100 mM) at 0.3 mL/min.

To concentrate the protein, if necessary, protein solutions were lyophilized to dryness, and the residual powder re-dissolved in the desired volume of water and dialyzed overnight. The protein concentration was determined by absorbance at 280 nm (ε_280_ = 5,120 M^−1^ cm^−1^), and the identification of the protein was confirmed by LC-MS analysis. Samples were stored at 4°C until use.

### EPR Spectrometry

Samples were prepared by mixing CuSO_4_ (final concentration 200 μM) with increasing amounts SUP. The sample volume was adjusted to 200 μL by addition of buffer (Tris-HCl, 10 mM, pH 7.5, 300 mM NaCl) and samples were incubated at RT for 2 h. Ten percentage glycerol was added, and samples were transferred into EPR tubes and flash-frozen in liquid nitrogen. X-band EPR spectra were recorded on a Bruker EMX spectrometer at 150 K using an ER-4102-ST rectangular cavity. All measurements were replicated on at least two independent samples, showing identical spectra.

### Fluorescence Spectrophotometry

A sample of SUP was diluted in HEPES buffer (20 mM, pH 7.4) containing 100 mM NaCl to a final concentration of 5 μM. Increasing amounts of a 1 mM stock solution of CuSO_4_ were added to the sample, and fluorescence emission spectra were immediately recorded on a Cary Eclipse spectrometer at RT (λ_exc_ = 260 nm, λ_em_ = 290–400 nm, slits 5 nm) using a 10 × 4 mm quartz cuvette. Recording spectra after various equilibration times (min to h) did not show any variation in signal intensity, indicating that the binding of Cu^2+^ was rapid. Data presented is the average of three independent titrations.

### Circular Dichroism

Circular dichroism spectra were recorded on a Jasco spectrophotometer using a 1 mm path length quartz cuvette. All measurements were made at room temperature. To a 30 μM solution of SUP in 10 mM Tris-HCl buffer, pH 7.5, containing NaCl 300 mM were added increasing amounts of CuSO_4_ (1 mM stock) or UO_2_(NO_3_)_2_ (0.414 mM stock). The solutions were carefully mixed to homogeneity before recording spectra. Spectra were recorded in triplicate and averaged.

To determine the K_d_ of SUP for ${\text{UO}}_{2}^{2+}$, a 30 μM solution of SUP and UO_2_(OAc)_2_ was titrated with increasing amounts of Na_2_CO_3_ and the change in ellipticity at 222 nm recorded. Titration was repeated in triplicates, showing consistent results. A representative titration curve was fitted using the program Dynafit (Kuzmič, 1996) as a competition between the formation of the ${\text{UO}}_{2}^{2+}$-SUP complex and the uranyl carbonate complexes: UO_2_(CO_3_), UO_2_${\left({\text{CO}}_{3}\right)}_{2}^{2-}$, and UO_2_${\left({\text{CO}}_{3}\right)}_{3}^{4-}$. The formation constants of the three carbonate forms were fixed at 1.45 × 10^−9^ M^−1^, 3.31 × 10^−16^ M^−2^, and 1.23 × 10^−22^ M^−3^, respectively, with the values being taken from reference (Zhang et al., 2014). The concentration of ${\text{CO}}_{3}^{2-}$ at each titration point was adjusted for ${\text{HCO}}_{3}^{-}$ formation by measuring the pH at each point and calculating the ${\text{CO}}_{3}^{2-}$ concentration using a pKa of 9.65 (Zhang et al., 2014).

## Data Availability

All datasets generated for this study are included in the manuscript/supplementary files.

## Author Contributions

MH, ZC, and EM conceived the study and designed the experiments. MH performed the experiments. MH, KK, ZC, and EM analyzed the data. MH and EM wrote the paper.

### Conflict of Interest Statement

The authors declare that the research was conducted in the absence of any commercial or financial relationships that could be construed as a potential conflict of interest.

## References

[B1] Adman E. T. (1991). Copper protein structures. Adv. Prot. Chem. 42, 145–197. 1793005 10.1016/s0065-3233(08)60536-7

[B2] Andreini C. Banci L. Bertini I. Rosato A. (2008). Occurrence of copper proteins through the three domains of life: a bioinformatic approach. J. Proteome Res. 7, 209–216. 10.1021/pr070480u 17988086

[B3] Banerjee R. Sheet T. (2017). Ratio of ellipticities between 192 and 208 nm (*R*_1_): an effective electronic circular dichroism parameter for characterization of the helical components of proteins and peptides. Proteins 85, 1975–1982. 10.1002/prot.25351 28707342

[B4] Barak Y. Ackerley D. F. Dodge C. J. Banwari L. Alex C. Francis A. J.. (2006). Analysis of novel soluble chromate and uranyl reductases and generation of an improved enzyme by directed evolution. Appl. Environ. Microbiol. 72, 7074–7082. 10.1128/AEM.01334-06 17088379 PMC1636143

[B5] Bhalara P. D. Punetha D. Balasubramanian K. (2014). A review of potential remediation techniques for uranium(VI) ion retrieval from contaminated aqueous environment. J. Environ. Chem. Eng. 2, 1621–1634. 10.1016/j.jece.2014.06.007

[B6] Brulfert F. Safi S. Jeanson A. Martinez-Baez E. Roques J. Berthomieu C.. (2016). Structural environment and stability of the complexes formed between calmodulin and actinyl ions. Inorg. Chem. 55, 2728–2736. 10.1021/acs.inorgchem.5b02440 26954703

[B7] Campbell A. L. Mangan S. Ellis R. P. Lewis C. (2014). Ocean acidification increases copper toxicity to the early life history stages of the polychaete *Arenicola marina* in artificial seawater. Environ. Sci. Technol. 48, 9745–9753. 10.1021/es502739m 25033036

[B8] Carugo O. (2018). Structural features of uranium-protein complexes. J. Inorg. Biochem. 189, 1–6. 10.1016/j.jinorgbio.2018.08.014 30149122

[B9] Chakraborty S. Kravitz J. Y. Thulstrup P. W. Hemmingsen L. DeGrado W. F. Pecoraro V. L. (2011). Design of a three-helix bundle capable of binding heavy metals in a triscysteine environment. Angew. Chem. Int. Ed. 50, 2049–2053. 10.1002/anie.201006413 21344549 PMC3058785

[B10] Garribba E. Micera G. (2006). The determination of the geometry of Cu(II) complexes: an EPR spectroscopy experiment. J. Chem. Educ. 83:1229 10.1021/ed083p1229

[B11] Handley-Sidhu S. Keith-Roach M. J. Lloyd J. R. Vaughan D. J. (2010). A review of the environmental corrosion, fate and bioavailability of munitions grade depleted uranium. Sci. Total Environ. 408, 5690–5700. 10.1016/j.scitotenv.2010.08.028 20858561

[B12] Kou S. Yang Z. Luo J. Sun F. (2017b). Entirely recombinant protein-based hydrogels for selective heavy metal sequestration. Polym. Chem. 8, 6158–6164. 10.1039/C7PY01206C

[B13] Kou S. Yang Z. Sun F. (2017a). Protein hydrogel microbeads for selective uranium mining from seawater. ACS Appl. Mater. Interfaces 9, 2035–2039. 10.1021/acsami.6b15968 28059497

[B14] Kuzmič P. (1996). Program DYNAFIT for the analysis of enzyme kinetic data: application to HIV proteinase. Anal. Biochem. 237, 260–273. 10.1006/abio.1996.0238 8660575

[B15] Le Clainche L. Plancque G. Amekraz B. Moulin C. Pradines-Lecomte C. Peltier G.. (2003). Engineering new metal specificity in EF-hand peptides. J. Biol. Inorg. Chem. 8, 334–340. 10.1007/s00775-002-0419-2 12589569

[B16] Le Clainche L. Vita C. (2006). Selective binding of uranyl cation by a novel calmodulin peptide. Environ. Chem. Lett. 4, 45–49. 10.1007/s10311-005-0033-y

[B17] Montes-Bayón M. Blanco-González E. (2016). Metalloproteins, in Metallomics: Analytical Techniques and Speciation Methods (Wiley-Blackwell), 339–357.

[B18] Newsome L. Morris K. Lloyd J. R. (2014). The biogeochemistry and bioremediation of uranium and other priority radionuclides. Chem. Geol. 363, 164–184. 10.1016/j.chemgeo.2013.10.034

[B19] Odoh S. O. Bondarevsky G. D. Karpus J. Cui Q. He C. Spezia R. (2014). UO22^+^ uptake by proteins: understanding the binding features of the super uranyl binding protein and design of a protein with higher affinity. J. Am. Chem. Soc. 136, 17484–17494. 10.1021/ja5087563 25411020

[B20] Peisach J. Blumberg W. E. (1974). Structural implications derived from the analysis of electron paramagnetic resonance spectra of natural and artificial copper proteins. Arch. Biochem. Biophys. 165, 691–708. 10.1016/0003-9861(74)90298-7 4374138

[B21] Plegaria J. S. Dzul S. P. Zuiderweg E. R. Stemmler T. L. Pecoraro V. L. (2015). Apoprotein structure and metal binding characterization of a *de novo* designed peptide, α3DIV, that sequesters toxic heavy metals. Biochemistry 54, 2858–2873. 10.1021/acs.biochem.5b00064 25790102 PMC4492461

[B22] Rubino J. T. Franz K. J. (2012). Coordination chemistry of copper proteins: how nature handles a toxic cargo for essential function. J. Inorg. Biochem. 107, 129–143. 10.1016/j.jinorgbio.2011.11.024 22204943

[B23] Starck M. Sisommay N. Laporte F. A. Oros S. Lebrun C. Delangle P. (2015). Preorganized peptide scaffolds as mimics of phosphorylated proteins binding sites with a high affinity for uranyl. Inorg. Chem. 54, 11557–11562. 10.1021/acs.inorgchem.5b02249 26583259

[B24] Van Horn J. D. Huang H. (2006). Uranium(VI) bio-coordination chemistry from biochemical, solution and protein structural data. Coord. Chem. Rev. 250, 765–775. 10.1016/j.ccr.2005.09.010

[B25] Wegner S. V. Boyaci H. Chen H. Jensen M. P. He C. (2009). Engineering a uranyl-specific binding protein from NikR. Angew. Chem. Int. Ed. 48, 2339–2341. 10.1002/anie.200805262 19199314

[B26] Xie Y. Chen C. Ren X. Wang X. Wang H. Wang X. (2019). Emerging natural and tailored materials for uranium-contaminated water treatment and environmental remediation. Prog. Mater. Sci. 103, 180–234. 10.1016/j.pmatsci.2019.01.005

[B27] Zhang C. Li C. J. He C. Liu J. Zhang L. Zhou L.. (2014). A protein engineered to bind uranyl selectively and with femtomolar affinity. Nat. Chem. 6:236. 10.1038/nchem.1856 24557139

[B28] Zhang X.-J. Wang X.-W. Da X.-D. Shi Y. Liu C. Sun F.. (2018). A versatile and robust approach to stimuli-responsive protein multilayers with biologically enabled unique functions. Biomacromolecules 19, 1065–1073. 10.1021/acs.biomac.8b00190 29443516

